# Harmonize rules for digital sequence information benefit-sharing across UN frameworks

**DOI:** 10.1038/s41467-024-52994-z

**Published:** 2024-10-09

**Authors:** Scarlett Sett, W. John Kress, Michael Halewood, David Nicholson, Genuar Nuñez-Vega, Davide Faggionato, Mathieu Rouard, Marcel Jaspars, Manuela da Silva, Christine Prat, Débora S. Raposo, Irma Klünker, Jens Freitag, Christian Keambou Tiambo, Carolina dos Santos Ribeiro, Linda Wong, Halima Benbouza, Jörg Overmann, DSI Scientific Network signatory authors, K. C. Bansal, Yiming Bao, Martha Lucía Cepeda-H, Solenne Correard, Sonigitu Ekpe, Desiree Hautea, Martine Hossaert-McKey, Takahide A. Ishida, Sally Katee, Harris Lewin, Ann M. Mc Cartney, Melania Muñoz-Garcia, Pablo Orozco, Michelle Rourke, Jens Sundström, Mutsuaki Suzuki, Rajeev Varshney, Elisa Vendramin, Ignazio Verde, Eizadora Yu, Maria Mercedes Zambrano, Amber Hartman Scholz

**Affiliations:** 1 CSIRO, Australian Centre for Disease Preparedness, Geelong, 3220 Australia; 2 Leibniz Institute DSMZ-German Collection of Microorganisms and Cell Cultures, 38124 Braunschweig, Germany; 3 National Museum of Natural History, Smithsonian Institution, Washington, DC 20013 USA; 4 Bioversity International, 00153 Rome, Italy; 5 Wellcome Sanger Institute, Cambridgeshire, CB10 1SA United Kingdom; 6 Bioversity International, 34397 Montpellier, France; 7 Marine Biodiscovery Centre, Department of Chemistry, University of Aberdeen, Old Aberdeen, AB24 3UE UK; 8 Oswaldo Cruz Foundation - Fiocruz Biodiversity and Health Biobank, 21040-361 Rio de Janeiro, Brazil; 9 Unite des Virus Emergents, Aix-Marseille University, 13005 Marseille, France; 10 German Federation for Biological Data (GFBio e.V.), 28359 Bremen, Germany; 11 Faculty of Law, Humboldt University of Berlin, 10117 Berlin, Germany; 12 Research Group Norm Setting and Decision Processes, Weizenbaum Institute, 10623 Berlin, Germany; 13 Leibniz Institute of Plant Genetics and Crop Plant Research (IPK), 06466 Gatersleben, Germany; 14 Centre for Tropical Livestock Genetics and Health, ILRI, Naivasha Rd, Nairobi, Kenya; 15 Netherlands National Institute for Public Health and the Environment, 3721 MA Bilthoven, Netherlands; 16 China Biodiversity Conservation and Green Development Foundation, Beijing, China; 17 Institut des Sciences Vétérinaires et sciences Agronomiques, Université de Batna, 05000 Batna, Algeria; 18 Technical University of Braunschweig, Braunschweig, Germany; 19 National Academy of Agricultural Sciences (NAAS), NASC Complex,, New Delhi, India; 20 National Genomics Data Center, China National Center for Bioinformation, Beijing, China; 21 Universidad Central, Bogotá, Colombia; 22 Wise Ancestors, Santa Cruz, California, United States of America; 23 Cross River State Ministry of Environment, Calabar, Nigeria; 24 University of the Philippines Los Baños, Los Baños, Philippines; 25 National Centre for Scientific Research – Institute of Ecology and Environment, Paris, France; 26 National Institute for Environmental Studies, Tsukuba, Onogawa Japan; 27 International Livestock Research Institute/CTLGH, Nairobi, Kenya; 28 Arizona State University, Phoenix, United States of America; 29 University of California, Santa Cruz, United States of America; 30 Law Futures Centre, Griffith University, Nathan, Australia; 31 Swedish University of Agricultural Sciences, Umea, Sweden; 32 National Institute of Genetics, Shizuoka, Japan; 33 Murdoch University, Perth, Australia; 34 Council for Agricultural Research and Economics (CREA) - Research Centre for Olive, Fruit and Citrus Crops, Rome, Italy; 35 University of the Philippines, Quezon City, Philippines; 36 Corpogen Research Institute, Bogotá, Colombia

**Keywords:** Policy, DNA sequencing

## Abstract

As multiple UN fora develop parallel rules for sharing benefits from digital sequence information, we urge better coordination. International policymakers should focus on harmonizing new benefit-sharing rules to ensure open access to data, database interoperability, and better benefit sharing outcomes.

Steven Spielberg’s *Life on Our Planet* begins with LUCA – the last universal common ancestor – a humbling reminder that all life shares a common origin, a universal code. This universality has fundamental implications for life scientists. With the decoding of DNA, the molecular blueprint for life, open infrastructures like public DNA sequence databases have become repositories for data from all life forms, from the microscopic to the macroscopic. Due to the universal nature of the DNA code, an interconnected, global dataset with maximal biological coverage became indispensable for understanding the fundamental biological processes that make life possible.

While scientists shared genetic data (or digital sequence information, DSI) from across the tree of life over the past four decades, laws on access to and utilization of (physical) biological resources were also developed. The 1992 UN Convention on Biological Diversity (CBD) underscored that countries have sovereign rights over their non-human biological resources and aimed to promote the fair and equitable sharing of benefits arising from the utilization of genetic resources (GR). In practical terms, countries were empowered to make national laws governing physical access to their genetic resources, to ensure benefits were returned to the provider country. In 2010, the CBD’s Nagoya Protocol created a legally binding instrument where each country may (but not must) establish conditions of use of its genetic resources and require users to share back with the provider country any monetary or non-monetary benefits that arise from the utilization of those resources.

Benefit sharing systems are also found in other UN fora such as the Food and Agriculture Organization’s International Treaty on Plant Genetic Resources for Food and Agriculture (ITPGRFA), the World Health Organization’s (WHO) Pandemic Influenza Preparedness (PIP) Framework and the under-negotiation Pandemic Agreement (WHO CA+), and the recently adopted agreement under the UN Convention on the Law of the Sea on the conservation and sustainable use of marine biological diversity of areas beyond national jurisdiction (BBNJ) treaty. Until recently, these agreements mainly focused on access to physical biological material.

The political realization that access and benefit-sharing (ABS) rules developed decades ago for access to physical genetic resources are often disconnected from access and use of open biological sequence data has placed the world of open data and its accompanying freely accessible global archive on a collision course with these UN fora. While some countries have issued national laws on the use of DSI^1^, use of open DSI has been unregulated at the international level. However, countries are now actively negotiating multilateral rules on how to share monetary and non-monetary benefits from the use of DSI in four relevant UN fora (i.e. CBD, ITPGRFA, WHO CA+ and BBNJ).

The development of these UN agreements, for the past 40 years, was driven by sectoral interests over different time points and has led to a patchwork of international laws and national implementing measures that create a labyrinth of rules and conditions for researchers to understand. While these UN fora are under no obligation to make rules that are cross-compatible for users of the global DSI archive, we currently have an opportunity for harmonization and futureproofing that we did not have in the past. We believe the relationship between the new DSI benefit sharing systems being developed under the four UN fora can be structured to increase benefit sharing and maintain open access and interoperability of DSI databases.

## Why do users of DSI need integrated access across biologically diverse datasets?

The power of DSI fully unfolds when comparison with all other sequences is possible. A single DNA or amino acid sequence is of little use and must be annotated and analyzed for similarities and differences to thousands of other sequences to fully exploit its scientific value, complemented by hands-on laboratory work. Research is inherently interdisciplinary and collaborative across sub-disciplines and sectors. Researchers are interested in natural relationships, rather than man-made divisions of the various UN fora, to answer critical scientific questions.

**Fig. 1:**
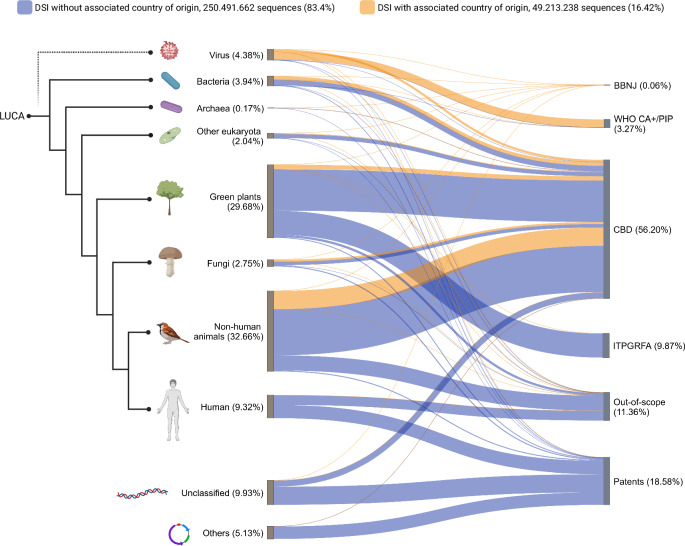
DSI is “mixed together” in a global dataset regardless of UN fora. Left side of the diagram: taxonomic distribution across 8 phylogenetic groups organized in a cladogram based on their phylogeny relative to the last universal common ancestor, LUCA and 2 groups without taxonomic information. “Virus” and “Other eukaryota” are non-monophyletic groups. The dashed and faded line connecting viruses to LUCA indicates uncertainties in the phylogenetic relationship of the group. The thickness of the lines connecting the two sides of the diagram is proportional to the number of sequences assigned to each taxonomic category and UN fora. Right side of the diagram: assignment of sequences to relevant UN fora based on absolute number of sequence entries in the database. DSI are color-coded indicating whether a country tag was explicitly indicated in the ENA metadata of each individual entry (yellow) as country of origin or if the tag was missing (blue). In parentheses, the percentage relative to the total dataset. See Supplementary Note 1 for details. Created in BioRender. Faggionato, D. (2024) BioRender.com/w04m281.

Open public DSI databases, such as the International Nucleotide Sequence Database Collaboration (INSDC), were built on these principles to facilitate, enable and maintain interoperability of data across domains of life to foster efficient and impactful analyses. The INSDC contains DSI generated from biological samples from nearly every existing environment across the planet. The data are “mixed up” together (Fig. 1) and available under the same open access conditions^2^.

If we assigned the data currently available in INSDC according to the four relevant UN agreements (assuming universal ratification and implementation and ignoring temporal scope) the greatest portion of the INSDC dataset comes from organisms within scope of the CBD and resulting national legislation (56%), followed by ITPGRFA crops (10%), WHO-relevant potentially-pandemic-causing pathogens (3%), and <1% to BBNJ (Fig. 1, Supplementary Note 1). Note that several industrialized countries do not require benefit sharing from the use of their genetic resources, but for the sake of simplicity, the data are included under the CBD scope. The “out of scope” category accounts for 11% and encompasses model organisms, synthetic sequences and human data. The “patents” category (19%) includes sequence reference material submitted as part of the patent application to ensure reproducibility of an invention under the World Intellectual Property Organization system (WIPO Standard 26). (WIPO agreed in May 2024 to require disclosure of country of origin for genetic resources and/or traditional knowledge in patent applications; however, a decision on whether this will could apply for DSI will be revisited in four years).

Fig. 1 demonstrates why harmonization is a significant issue for DSI users (i.e. scientists). Users might be interested in a specific group of animals or bacteria or a specific function like photosynthesis or cell signaling. Exploring those topics requires use of DSI from many fora and, in the future, compliance with all international rules on DSI. If those rules are complicated or unharmonized, that scientific work will be hindered.

To demonstrate the integrative manner of DSI analysis needed to address urgent scientific and societal questions, we have generated public health, food security and sustainable use case studies based on published literature that show how and why DSI users need integrated access to DSI from various UN fora (Box 1). These case studies reflect the way research is conducted, providing valuable lessons for policymakers to take into consideration moving forward in the negotiations.

Box 1 Human health, Food security and Sustainability case studies show how DSI from more than one UN fora is used to answer scientific questions **(A) Human health: Using DSI to develop a vaccine (WHO CA** + **& CBD/NP**)

The development of a COVID-19 vaccine was a complex procedure dependent on global scientific collaboration and open access to DSI. Moderna’s patent US-10702600-B1: betacoronavirus mRNA vaccine^5^, filed in February 2020, describes an mRNA vaccine for respiratory viruses, particularly betacoronaviruses (the genera that includes SARS-CoV-2, which causes COVID-19).

The patent draws on 176 publicly available (via INSDC) DSI from different respiratory viruses, from a large range of countries (e.g., Saudi Arabia, UK, UAE, Jordan, France, USA, Qatar, Thailand, Oman, China). It also discloses 96 new sequences, submitted to public DSI databases alongside the patent. Some are similar or identical to existing public sequences, others represent engineered or modified versions of sequences already referenced elsewhere in the patent document. Many are labelled as “Artificial Sequence” and have no country of origin. Not one SARS-CoV-2 sequence is listed in the patent. This large variety of sequences, including the omission of the specific SARS-CoV-2 sequences, demonstrates that Moderna was free to decide which sequences to include in the patent document, as long as they fulfilled the requirements for reproducibility. No single sequence was vital to its work. Interestingly, the mRNA present in the vaccine is only 70% identical to any natural SARS-CoV-2 found in the INSDC^2^.

**(B) Food security: Emerging crop resistance to new biological and environ-mental threats (CBD/NP & ITPGRFA)**

An estimated 70% of wheat (*Triticum aestivum)* produced around the world is used for human consumption and is an indispensable food source for one-third of the world´s population, especially as global populations continue to increase^6^. Although the ‘Green Revolution’ increased crop yields through breeding technologies, wheat crops are still susceptible to diseases. To date, investments in fungicides in Europe account for ∼€1.3 billion, 70% towards a single pathogen, *Zymoseptoria tritici*^7^, which causes Septoria tritici Blotch (STB) disease. As a result of widespread fungicide application, resistant *Z. tritici* strains have recently evolved.

A French study^8^ investigated microbial communities of wheat varieties, two susceptible to STB and one resistant strain, over a growing season. Using specialized domain-specific (not organism specific) open access DSI databases (e.g., SILVA^9^, UNITE^10^), researchers identified and compared fungal and bacterial communities in infected and healthy wheat to infer whether specific fungal/bacterial composition could act as beneficial biocontrol against *Z.tritici*. While *Z. tritici* was present in all samples, it showed higher abundance in unhealthy leaves and was less abundant on the variety that carried a known fungal resistance gene (i.e. Stb16q). The results showed that sampling date, wheat variety and health of the leaves had significant effects on the fungal and bacterial communities. The DSI used in this study was from crop genetic resources regulated under the ITPGRFA’ s multilateral system, and from weeds, fungal, and bacteria genetic resources regulated under national measures implementing the CBD/NP.

**(C) Sustainability: DSI databases used to develop sustainable, eco-friendly, and efficient fabric detergents based on cold-active enzymes (CBD/NP & BBNJ DSI)**

The production of efficient industrial enzymes, needed for waste reduction, energy efficiency, and even mitigation of greenhouse gases, is a central goal of the white biotechnology sector. Cold-active enzymes, from microorganisms living in temperature ranges of −2 to 20 °C can be found in a variety of environments, including deserts, mountains, wetlands, polar regions, and the deep-sea. These enzymes provide similar functionality at lower temperatures compared to their high-temperature counterparts.

In 2018^11^ a novel α-amylase (amy175) was characterized from a newly isolated Antarctic Sea ice bacterium (M175). The bacterial species identity was determined by comparing 16S rDNA gene sequences from INSDC and conducting phylogenetic analysis. The new species demonstrated high levels of similarity to 15 known species of *Pseudoalteromonas*, a species with widespread global distribution. Furthermore, the comparison of amy175 to 27 selected amylases DSI sequences confirmed that amy175 had highly conserved regions associate to amylase activity. While those 27 sequences provided valuable information to classify enzymatic activity none had country of origin information. The comparison of new DSI to previously available DSI in public databases is a critical tool for characterizing genetic material from novel organisms.

### Lesson #1: Ensure DSI interoperability

If DSI access or database interoperability is impeded, it will undermine research innovation rather than supporting and enabling it. For example, under the WHO CA+ discussions, negotiators have looked at the PIP Framework and have discussed a similar model for the new treaty. Influenza DSI and, more recently, SARS-CoV-2 DSI have often been deposited in the Global Initiative on Sharing All Influenza Data (GISAID) database, which curates and regulates access to these data. GISAID provides free-of-charge access to registered and verified accounts. In exchange, users must agree to a data access agreement including to acknowledge the submitting scientist when re-using data in a publication. Importantly, the agreement prohibits GISAID from integrating and sharing its data with fully open databases, such as INSDC (although the original data providers may share their data outside of GISAID). The vaccine development case study (Box 1A) would not have been possible if the only legally-certain source of viral DSI was in GISAID. Vaccine development requires exploring all of viral biodiversity, which is only available in the INSDC. It also requires the integration of DSI with other biological protein sequence data and structures for vaccine targets (epitopes) and the selected target’s variation and molecular structure. DSI is combined with chemical and enzymatic knowledge to increase stability and ensure proper folding. To develop pandemic-responsive research and products, DSI needs to be integrated with many types of viral DSI (and the respective databases that house these data) including human data (to understand host-pathogen relationships), protein structure data (to move towards vaccine development), or clinical and imaging data (for new ideas on disease progression). A single, closed, non-interoperable database will not enable pandemic preparedness.

### Lesson #2: Make benefit-sharing rules as similar as possible

Most users will not know which UN fora the DSI they are using could belong to because research questions are driven by a need to understand biological processes and not based on the origin of DSI (geographical or legal provenance). If each UN forum develops its ‘own DSI rules’, for example, on when, how, or whether to report or share benefits, then users will need to track and trace the use of thousands of sequences used and engage with many different platforms and administrative activities. However, if similar triggers for aggregate DSI benefit sharing could be established in all UN fora, this would minimize the risk of avoidance, incentivize and encourage compliance, and obliviate the need to track and trace^3^.

### Lesson #3: Only harmonized DSI benefit-sharing rules will capture benefits from artificial DSI

With synthetic biology advancements, a dis-harmonized approach to DSI benefit-sharing could incentivize DSI mechanism avoidance by synthesizing new sequences that cannot be traced back to a geographical or legal provenance. Either doing this through codon optimization or artificial intelligence (AI), it would be nearly impossible to trace back to the original nature-based DSI because the new synthetic sequence would be quite different. For example, for the sustainability case study (Box 1C), the next step in biotech development of this cold-tolerant enzyme is to further synthesize and optimize the enzymatic properties. For-profit entities will create consensus sequences to take the most common (genetically conserved) amino acid at every position in the enzyme and create new, synthetic enzymes. Synthetic sequences are human-made products that pool DSI derived from genetic resources regulated under multiple fora. Thus, they cannot be tracked back to a single country or jurisdiction of origin. A similar issue would arise for the vaccine case study (Box 1A), where the final sequence used in the vaccine was only 70% similar to any natural sequence deposited in the database. With harmonization across all relevant fora, all rules and roads can convergently lead to DSI benefit-sharing, ideally aggregated across use of any and all DSI, no matter how novel or man-made DSI might become.

## Options for “harmonious” DSI benefit sharing rules across UN fora

As demonstrated above, to maximize the societal value of DSI, it must be made available in the most integrated and interconnected way possible across all species and jurisdictions. While the international rules on DSI benefit-sharing are all still under negotiation, the risk of fragmentation is inevitable if policymakers assume DSI and its outcomes can be neatly separated into siloes. But, what can policymakers do to create an enabling environment? Here we present various “house models” where the house is a placeholder for the concept of DSI benefit-sharing and not database infrastructures. Doors indicate triggers in which new monetary benefits will be required but are not intended to imply a paywall (or a closed access) approach to DSI benefit-sharing as these would severely limit benefit-sharing^3,4^. Windows indicate how the monetary benefits will be delivered to the relevant UN fora.

## Option 1: One house with one door and four windows

**Fig. 2:**
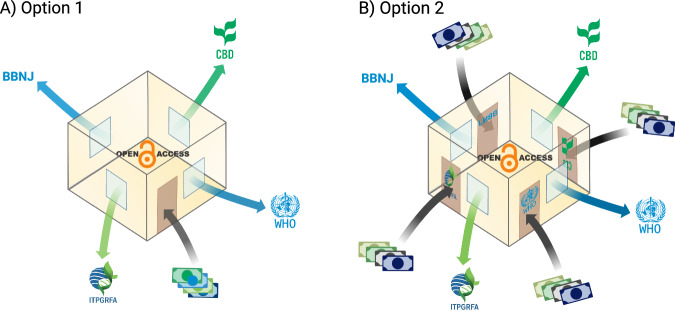
Two proposed models for harmonization between UN fora. **A** Option 1 - One house with one door and four windows. **B** Option 2 - One house with four doors and four windows. Inside the house lies the open database ecosystem with the DSI of relevance to all different UN fora without jurisdictional sub-division. Doors indicate trigger points for monetary benefits and windows indicate how the monetary benefits will be delivered to the relevant UN fora. The house represents the DSI benefit-sharing mechanism and not database infrastructures. Created in BioRender. Internationalisation, S. (2024) BioRender.com/h63v844 (A) and BioRender.com/u09u989 (B).

If one were to design a monetary benefit sharing system for DSI from scratch, a single global fund that collected benefits from any type of use of DSI would be a natural choice. In option 1 (Fig. 2A), all four UN fora would adopt identical triggers for payment and soft law norms for DSI governance. All payments by all users would be made to a single existing global fund (1 door) such as the Global Biodiversity Framework fund (or another appropriate global facility), which would distribute funds (through four windows) to the UN instruments for further disbursement according to their respective governance procedures. Distribution from the global fund to each of the four UN fora could be proportional to the payments made by users in the relevant sectors or informed by the quantity of respective DSI available in open access databases (Fig. 1). However, this option is politically difficult as it requires all four UN fora to adopt identical benefit sharing conditions and the use of one single global fund. The ITPGRFA, for example, already has a multilateral ABS system, which provides a useful basis upon which to ‘layer’ obligations for DSI benefit sharing, thus migrating to a CBD-led DSI benefit-sharing process is not particularly attractive. The WHO pathogen Access and Benefit Sharing System will likely keep monetary benefits within the WHO system to fund pandemic prevention and preparedness efforts. However, for BBNJ DSI, because it is a very small dataset (less than 1%), a not-yet-established mechanism, and thematically focused on biodiversity, it is easier to imagine it could join forces a priori with the CBD multilateral mechanism.

## Option 2: One house, four doors and four windows

For option 2 (Fig. 2B), each fora would have separate benefit sharing funds, receiving payments directly from users (through separate doors), and disbursing funds according to priorities established by the fora’s governance mechanisms (through four windows). In this model, a benefit sharing payment made through any door to any of the four instruments would fulfill benefit-sharing obligations in all of the others and ensure simplicity and freedom to operate. Similarly, non-monetary benefits shared and reported in a global clearinghouse would fulfill obligations in all of the others.

**Fig. 3:**
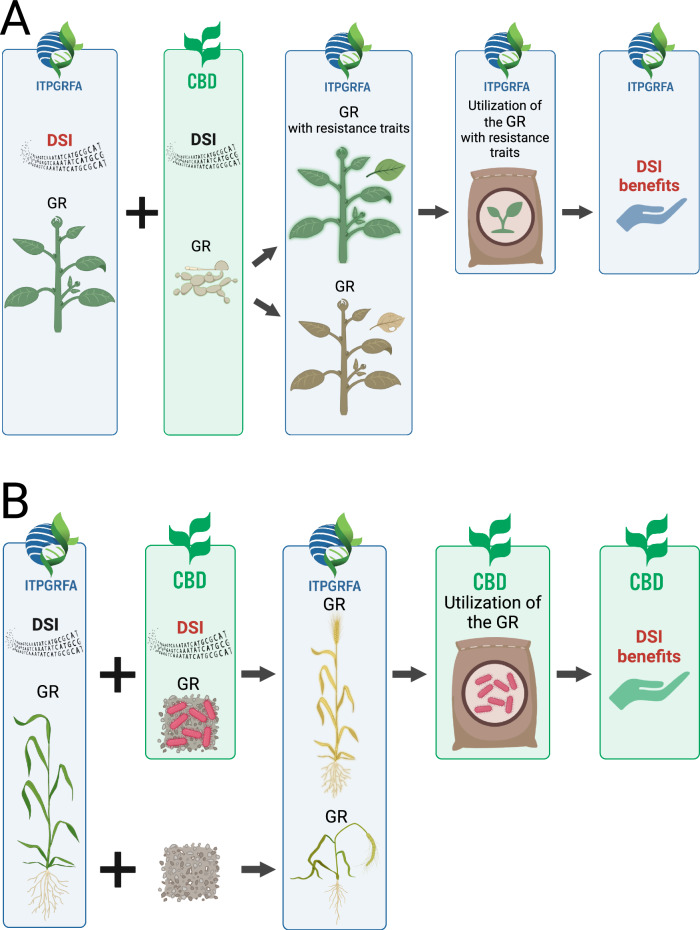
Flowcharts of two possible scenarios for benefit-sharing distribution under Option 2. Scenario A: development of a new variety of an ITPGRFA Annex I plant that is resistant to a fungus that falls under the CBD. Although DSI of both the Annex I plant and the CBD-relevant fungi have been used, the DSI-related benefits resulting from the use of the new plant variety will stream to the ITPGRFA fund. In scenario B: identification of a bacterial strain in soil that falls under the scope of the CBD that promotes the growth of an ITPGRFA Annex I plant under stress conditions. Although DSI of both the bacterium and the plant have been instrumental to the discovery, the DSI-related benefits generated by the use of the bacteria strain will stream to the CBD fund. DSI Digital Sequence Information, GR physical Genetic Resource. Created in BioRender. Faggionato, D. (2024) BioRender.com/m52p511 (**A**) and BioRender.com/u21i840 (**B**).

Under option 2, the four UN fora do not need identical benefit sharing triggers, but they do all need to require payments in a similar way (e.g., a proportion of aggregate sales of products at the end of value chains). They also need to reciprocally recognize that benefit-sharing payments under any of the other four fora qualifies users to access all DSI without needing to calculate the proportion of DSI from different biota that were used throughout the research and development process. For example, the sale of seeds of a new pest resistant Annex 1 plant variety would trigger payments to only the ITPGRFA benefit-sharing fund, even if the DSI of pathogens were used as part of the research to develop the variety (Fig. 3A). Conversely, the sale of a new biocontrol agent, e.g. a soil bacterium, which required using DSI from ITPGRFA Annex 1 crops, would trigger benefit sharing payments (only) to the CBD fund (Fig. 3B).

For both options, users would continue to have open access to all publicly available DSI as long as they complied with benefit-sharing requirements and relevant monetary triggers to any (one) of the four UN fora. Both options would ensure open, integrated access to all public DSI; simplicity and legal certainty for users; no incentives for forum shopping between systems and no need for tracing/tracking from which fora the used DSI came from. It would also allow for benefit-sharing from the use of artificial sequences (i.e. produced by consensus sequence or increasingly AI) because origin would not be the determining factor for payment. The simplicity of either option for harmonization would be lost if one or more of the UN fora adopts a benefit sharing formula that involve tracking the proportional contribution of different DSI to the development of products. Under a dis-harmonized system, claims for benefit sharing from one UN fora or one country to a sequence that is only partially (X %) similar to a natural one would be difficult to make. If there is no harmonization, users of DSI could find or create sequences with fewer benefit-sharing obligations.

## Summary

A harmonized multilateral system for DSI should have clear and simple standardized conditions across fora for the use of all publicly available DSI. Box 2 lays out recommendations from members of the scientific community that would incentivize users to comply with obligations and improve legal certainty and clarity. For the research community, an extraordinarily important ‘whole’ must be preserved, nurtured, and expanded by ensuring DSI is open access, interoperable, and universally accessible. Options 1 and 2 above demonstrate that it is possible to develop new benefit sharing rules for DSI that build on the institutional infrastructures of the CBD, Plant Treaty, WHO and BBNJ without dividing the governance of the global DSI data set between sectoral lines.

Harmonization among the different frameworks will undoubtedly be challenging, as each framework serves a different purpose, including the conservation and sustainable use of biodiversity (CBD/NP/BBNJ), disease detection, prevention, and eradication (PIP, WHO CA+), and food security (IPTGRFA). Each framework has different decision-making processes, compliance measures, and designated national negotiators (often from different government ministries) with mandates that may not overlap or even compete for budgets and political prioritization. However, if benefit-sharing from DSI for each of these fora is conceived of separately rather than in an interlinked global context, a real risk exists that the value of these data will decrease due to legal uncertainty and bureaucratic burden. Ironically, the new DSI system that aims to maximize benefits would instead reduce the benefits being created.

Research is the primary pathway through which benefits from DSI are realized. Benefits (monetary and non-monetary) come in a range of forms, such as generating new knowledge and the development of scientific innovations. An opportunity to develop a harmonized mechanism(s) for the use of DSI that is compatible with scientific practices and DSI database structures, while at the same time maximizing benefits shared from the use of DSI to help achieve the objective of individual frameworks, is now before us.

Box 2 Recommendations to enable DSI harmonization

1. ***Cross-recognize DSI benefits shared.*** As proposed in Option 2, any benefit sharing payments made through any “door” to any of the four UN instruments should be recognized as fulfilling benefit-sharing obligations in all of the others to ensure simplicity and freedom to operate. Similarly, non-monetary benefits shared and reported in a global clearinghouse would fulfill obligations in all of the others.
2. ***Create an inter-fora expert advisory board*** that supports the process to develop harmonized rules between the four UN fora so that benefit sharing contributions in one system can be recognized in the other three. If users benefit-sharing obligations arise under just one system, they should nonetheless have access to the entire global DSI dataset. Engaging with a broad range of stakeholders (policymakers, academia, database managers, private sector and indigenous peoples and local communities) through informal dialogue and inter-forum discussion will enrich the decision-making process and potential outcomes^12^. Users of DSI are often the ones directly impacted by the rules and restrictions imposed by these international frameworks and cannot be isolated from the decision-making process on benefit-sharing. Active engagement from the academic sector during the development of the DSI multilateral mechanism has been essential to inform the process and provide empirical evidence on how DSI data is created, used, and shared. It is critically important that negotiators consider the broader picture of how all four agreements can work together, in mutually supportive ways, to contribute to a whole greater than the sum of the four parts.
3. ***Learn how harmonization has been achieved in other UN processes****.* Other global decision-making bodies have harmonized their procedures across multiple fora (e.g., customs export control, international air transport association, weather data and model forecasting, etc.) and can serve as examples of how this result can be achieved, especially with regards to scientific and technical issues. We suggest that the CBD commission a study on how harmonization in other UN processes has been achieved in the past and how those processes can be managed to reach harmonization. This can provide lessons for a DSI inter-fora system. This could be a first step in working together towards a common goal.
4. ***Signal to other fora the desire to work together on DSI***. There are simple signals negotiators can send to indicate the intent that rules on benefit-sharing they create in one forum to work integratively with rules in the other fora. For example, using the same (or lack thereof) definition for DSI, sufficiently similar trigger points for when benefits should be shared, textual reference and citations of other instruments, all can work together to signal to users and lawyers alike that the DSI rules are intended to integrate across the scientific DSI ecosystem rather than to fragment it.
5. ***Develop a comprehensive global DSI capacity-building and non-monetary benefit-sharing plan****.* There remain significant gaps in global capacities to produce and use DSI. All four UN fora indicate their intention to invest in DSI-related capacity strengthening but few efforts have been made to define how and what that would be, and no efforts have been made to develop an ambitious, global plan to truly impact inequalities in DSI use. For example, regional centers of excellence with different sectoral focuses (health, agriculture, bioeconomy, conservation) could each be led by different fora but share core features such as training, infrastructure development, cloud services, in cross-cutting areas. A harmonized, global DSI capacity building strategy that would complement efforts and integrate with existing portals such as UNESCO’s The World Academy of Sciences, could create concrete, impactful actions on DSI outcomes. For non-monetary benefits, a harmonized approach to reporting and monitoring DSI non-monetary benefits across all UN fora would be vastly simpler than multiple rules, mechanisms and portals.

## Supplementary information

Supplementary Information
