# Supplementary material for: Harmonize rules for digital sequence information benefit-sharing across UN frameworks
Source: Nat Commun. 2024 Oct 9;15:8745. doi: 10.1038/s41467-024-52994-z (PMC11464523; doi:10.1038/s41467-024-52994-z)
Supplement: Supplementary file 1 — Supplementary Information [file 41467_2024_52994_MOESM1_ESM.pdf]

**Supplementary Note 1**

**Harmonize rules for digital sequence information benefit-sharing across UN frameworks**

## 1. Overview

Data used for Figure 1 originate from the European Nucleotide Archive (ENA) database ( https://www.ebi.ac.uk/ena/portal/api/search?result=sequence&limit=0&format=tsv&download=true) downloaded on the 27th of April 2024 and represent metadata from 299,704,900 ENA accessions.

The categorization of individual sequences into the six categories (right side of Figure 1) was based upon a variety of metadata properties and legal understanding. For two of the UN groups (WHO CA+/PIP and ITPGRFA), categorization was based on the taxonomic identity of the Genetic Resource (GR) from which the nucleotide sequence, or Digital Sequence Information (DSI), originated. For the BBNJ treaty, the geographic origin (specifically international waters without additional country information) associated with the individual entry was used to place sequences in the BBNJ category (Figure 1, right-hand side). Sequences originating from humans, model organisms, and synthetic/chimeric sequences were grouped into the "Out-of-scope" category based upon current legal understanding of material scope (see point 2.3 for more details). Accessions associated with patents, regardless of their taxonomic affiliation or geographic origin, were grouped in the Patent category based upon the presence of the **/PAT** tag (https://www.ebi.ac.uk/patentdata/nucleotides). These sequences are not necessarily the object of the claim of intellectual property *per se*, but are often reference material submitted as part of a patent application to ensure reproducibility of the patented invention. All remaining sequences were categorized under CBD as this Treaty has an otherwise all-encompassing geographical scope. However, temporal scope was not considered.

## 2. Detailed analysis of the assignment to various UN frameworks associated with each DSI accession

### 2.1. ITPGRFA

In order to classify the accessions associated with the ITPGRFA (Figure 1, right side), Annex I of the International Treaty on Plant Genetic Resources for Food and Agriculture (ITPGRFA) was used. Annex I includes a list of crop and forage plants that are covered by its multilateral system. The WiLDSI project mapped the organisms from Annex I to the NCBI taxonomy (https://github.com/wildsi/wildsi/tree/main/data/annex). The annotation was done manually by Andrew Hufton during the WiLDSI project (https://wildsi.ipk-

gatersleben.de/apex/wildsi/r/wildsi/home), including assignment of subtaxa and manual curation of each exception present in Annex I. In this study, we used the list created by the WiLDSI project in December 2021 "as is." For ITPGRFA accessions, we also observed that although 84% of ENA accessions have no **/country** metadata tags and thus cannot be associated with countries of origin in our analysis, it is possible to trace the country of origin by consulting the original publication where the accession was originally published. To note, the tag **/country** will be replaced by the tag **/geographical_origin** from June 2024 (https://ncbiinsights.ncbi.nlm.nih.gov/2023/12/14/update-genbank-qualifier/.).

### 2.2. WHO CA+/PIP

To identify the accessions associated with the "WHO CA+/PIP" group (Figure 1, right side), the information available in Annex 2 (https://www.who.int/publications/m/item/annex-2-of-the-international-health-regulations-(2005)) of the International Health Regulations (2005) (IHR), and from the WHO R&D (https://www.who.int/observatories/global-observatory-on-health-research-and-development/analyses-and-syntheses/who-r-d-blueprint/who-r-d-roadmaps) and WHO PIP (https://apps.who.int/gb/pip/pdf_files/pandemic-influenza-preparedness-en.pdf) plans were used.

#### 2.2.1 IHR

IHR defines countries rights and obligations in handling public health events that have the potential to cross borders. Under the IHR, States Parties are required to carry out an assessment of public health events occurring within their territories utilizing the decision instrument (algorithm) provided in Annex 2 of the Regulations (*Decision instrument for the assessment and notification of events that may constitute a public health emergency of international concern*). The annex identifies three possible events involving different diseases, the pathogens listed in each of the three events were used to identify taxonomic units from which to map the tax_id to be associated with the group "WHO CA+/PIP":

1. A case of the following diseases is unusual or unexpected and may have serious public health impact, and thus shall be notified

- Smallpox
- Poliomyelitis due to wild-type poliovirus
- Human influenza caused by a new subtype
- Severe acute respiratory syndrome (SARS)

2. Any event of potential international public health concern, including those of unknown causes or sources and those involving other events or diseases than those listed in the box on the left and the box on the right shall lead to utilization of the algorithm

- "Disease X" (placeholder for an unidentified or hypothetical disease).

NOTE: This cannot be defined taxonomically and so was not included in the analysis.

3. An event involving the following diseases shall always lead to utilization of the algorithm, because they have demonstrated the ability to cause serious public health impact and to spread rapidly internationally

- Cholera
- Pneumonic plague
- Yellow fever
- Viral haemorrhagic fevers (Ebola, Lassa, Marburg)
- West Nile fever
- Other diseases that are of special national or regional concern, eg. dengue, Rift Valley fever, and meningococcal disease.

### 2.2.2 WHO PIP

With regard to influenza-related taxonomic units, we focused our analysis on the units attributable to influenza type A, since only influenza type A viruses are known to have caused pandemics.

### 2.2.3 WHO R&D

The remaining human pathogens for taxonomic categorization within the WHO CA+/PIP category were collected from the global WHO R&D strategy (https://www.who.int/observatories/global-observatory-on-health-research-and-development/analyses-and-syntheses/who-r-d-blueprint/background), a list of diseases that require priority attention because they have epidemic potential. The list consists of the following diseases:

- COVID-19
- Crimean-Congo haemorrhagic fever
- Ebola virus disease and Marburg virus disease
- Lassa fever
- Middle East respiratory syndrome coronavirus (MERS-CoV) and severe acute respiratory syndrome (SARS)
- Nipah and henipaviral diseases
- Rift Valley fever
- Zika
- Monkeypox
- "Disease X"

The following methodology was used to map individual pathogen to the corresponding tax_id:

a. **Identify Virus Names associated with each disease**. This information was collected from Wikidata (https://www.wikidata.org/). For example, for Lassa fever, one of the most common virus name used is "Lassa virus." https://www.wikidata.org/wiki/Q706845.

b. **Confirmation and verification of the correspondence between if virus name and taxonomic name** (including old taxonomic names). Once the vernacular name of the disease was obtained, the taxonomic name was sourced from Wikidata and ICTV taxonomy (https://ictv.global/taxonomy). For example, "Lassa virus" was updated to "*Mammarenavirus lassaense*" in 2022: https://ictv.global/taxonomy/taxondetails?taxnode_id=20131157&taxon_name=Lassa%20virus.

c. **Identification of NCBI tax_id for each viral entity.** Next, the scientific names from the ICTV taxonomy were used for a text search in the NCBI taxonomy Browser (https://www.ncbi.nlm.nih.gov/taxonomy) to retrieve the corresponding tax_id. In case of an updated scientific name, the updated entry from the ICTV taxonomy database and any obsolete entry from the NCBI taxonomy Browser were mapped the corresponding tax_id. This allowed retrieval of any ENA accession that may be associated to obsoleted taxonomic description.

d. **Fetching of sub-tax_id**
All sub-tax_id hierarchically associated to higher level pathogen tax_id were retrieved with TaxonKit (Shen et al. 2021) using the higher taxonomic units as queries.

e. **Assignments of pathogen-associated ENA accessions to the WHO CA+/PIP group**
All tax_id collected in steps c and d, were assembled in a list and the list was used to categorize all entries in the ENA database associated to the "WHO CA+/PIP" group (Figure 1, right side).

### 2.3. Out-of-scope category

All synthetic or chimeric sequences not under the scope of any of the four UN fora, or associated to a patent, were considered “out-of-scope” (Figure 1, right side). Additionally, all organisms listed as model organisms from the NCBI website (https://www.ncbi.nlm.nih.gov/Taxonomy/Browser/wwwtax.cgi), except *Oryza sativa* and *Zea mays* (included in the ITPGRFA group) and *Homo sapiens* (isolated as its own group), were categorized into the "out-of-scope" group (Figure 1 right side):

1. *Arabidopsis thaliana*
2. *Escherichia coli*
3. *Pneumocystis carinii*

4. *Bos taurus*
5. Hepatitis C virus
6. *Rattus norvegicus*
7. *Caenorhabditis elegans*
8. *Saccharomyces cerevisiae*
9. *Chlamydomonas reinhardtii*
10. *Mus musculus*
11. *Schizosaccharomyces pombe*
12. *Danio rerio* (zebrafish)
13. *Mycoplasma pneumoniae*
14. *Takifugu rubripes*
15. *Dictyostelium discoideum*
16. *Xenopus laevis*
17. *Drosophila melanogaster*
18. *Plasmodium falciparum*

### 2.4. Patents category

All sequences that had the dataclass category mapped with the string PAT were associated with the category "Patents" (Figure 1, right-hand side).

### 2.5 BBNJ assignment based on geographic information

Assignment of accessions to the BBNJ group (Figure 1 right hand side), was done from the information contained in the metadata field country and location, according to the following principles:

- **Identification of a recognized ocean name with no reference to a country name**: In case the country metadata field mention only the string one of the ocean names without reference to a country, the accession is categorized in the BBNJ group., e.g. "Atlantic Ocean:Mid-Atlantic Ridge". A full, curated list of recognized ocean names is available at https://github.com/wildsi/wildsi/tree/main/data/country.

- **Identification of 'Ocean', 'sea' or 'bay' with reference to the name of a country :** In case the location metadata field mention the term "ocean" "sea" or "bay" and an explicit reference to the name of a country name in the country metadata field, the accession is considered to be "associated with a country of origin" and therefore not assigned to the BBNJ group.e.g. “USA:Hawaii, Oahu Island, Kaniohe Bay”. This is a less rigorous filter that enriches DSI data from coastal marine environments.

Following this methodology, we attempted to assign ENA accessions to the BBNJ treaty, aware that the data we extracted are an overestimate of the accessions that could potentially be associated with

the BBNJ group relying solely on well curate maritime information from the country:location metadata fields of the ENA database. In addition, precedence was given to the information contained in the country:location metadata fields over the tax_id with regard to assignment to the BBNJ group which may lead to inconsistencies in annotations, such as in the following spurious examples:

- ENA accessions with country:location metadata with information as such Mediterranean sea, North sea, Japan Pacific Ocean, Mediterranean sea: Croatia, Baltic sea were assigned to the BBNJ group, but it isn't certain that the ENA accessions were obtained from areas beyond national jurisdiction rather than continental waters.
- Accessions from the Mediterranean area that according to taxonomy refer to the ITPGRFA, but having the country:location metadata as Mediterranean_sea, are grouped in the BBNJ group, e.g., accession KY673694.1 of *Medicago truncatula*

For any other accessions that were not assigned to the BBNJ following the procedure described above, if the country field contained a text string, they were categorized as "DSI with associated country of origin," otherwise they were categorized as "DSI without associated country of origin" (green and red lines in Figure 1, respectively) and assigned to BBNJ.

**2.6. CBD category**

Accessions that did not fall into any of the other five categories, ITPGRFA, WHO CA+/PIP, BBNJ, Out-of-scope and Patents, were mapped to the CBD, (Figure 1, right-hand side).

**3. Retrieval of the nucleotide sequences and the metadata associated with each entry**

Each unique Accession id (Unique sequence id) was downloaded as tab-separated values (.tsv) using the ENA API (https://www.ebi.ac.uk/ena/portal/api/) together with the following associate metadata:

- country = "locality of isolation of the sequenced sample indicated in terms of political names for nations, oceans or seas, followed by regions and localities", e.g. country="Canada:Vancouver". For this analysis, country will be split into two fields, country and location.
- dataclass = "Sub-dataset of the Nucleotide sequences dataset. e.g. PAT = Patents"
- scientific_name = "lowest taxonomic unit associated to the GR from which the nucleotide sequence originates"
- tax_division = "Broader artificial taxonomic division, e.g. FNG = fungi"
- tax_id = "Unique taxonomic ID"

**4. Data analysis tools used**

The data was analyzed using the open-source computing framework Apache Spark (Zaharia et al., 2016) and its Python API PySpark (Apache Spark version 3.4.1) and TaxonKit (Shen et al. 2021). The Sankey diagram was generated with SankeyMATIC (https://sankeymatic.com/) and edited with BioRender (https://www.biorender.com/).

The figures have the following BioRender publication and licensing rights agreement numbers: Figure 1: BM27AR1OK9. Figure 2, option 1: VC27AJWUKN, Figure 2, option 2: ZW27AJX1BD, Figure 3, example A: YT27AKILGH, Figure 3, example B: QC27AKIGM4.

**5. Taxonomic identification of nucleotide sequences**

Taxonomic analysis followed taxonomic standardization implemented by NCBI and adopted by the International Nucleotide Sequence Database Collaboration (INSDC); the foundational initiative that operates between DDBJ, EMBL-EBI and NCBI (Federhen, 2012; Sakamoto & Ortega, 2021). ENA requires every entry in the database to possess a univocal taxonomic ID under the tax_id category that define the exact taxonomical unit (mostly genus and species) associated to the GR from which the entry originates. For example, each DSI accession belonging to rice (*Oryza sativa)* in the ENA database is associated to a unique numerical identifier that characterizes the genus and species of the genetic resource of origin (e.g. for *Oryza sativa,* tax_id = 4530). Tax_id allows therefore for the precise taxonomic categorization of each nucleotide sequence. However, many of the organisms included in Annex I of the ITPGRFA or the WHO CA+/PIP are not univocally identified at the species level, but rather at the genus or family level.

The hierarchical categorization of taxonomies from NCBI allowed for the retrieval and identification of taxonomic subunits cascading down from the higher levels to the lowest, to accurately capture the affiliation of each nucleotide sequence to each of the treaties and conventions, taking advantage of the NCBI taxonomic database, downloaded from NCBI's FTP servers (https://ftp.ncbi.nih.gov/pub/taxonomy/).

For example, in order to curate the list of species associated with Annex I of the ITPGRFA, dump files nodes.dmp and names.dmp containing the taxonomic information were used to complete the taxonomic tree below each taxon using python customized methods. For example, for *Oryza sativa* it's was possible to obtain its "*children"* taxa as follows:

- *Oryza sativa* (tax_id)
- *Oryza sativa Indica Group* (tax_id)
- *Oryza sativa aus subgroup* (tax_id)
- *Oryza sativa indica subgroup* (tax_id)

The taxonomic groupings on the left side of Figure 1 were created using TaxonKit (Shen et al. 2021) using the higher taxonomic units described by Schoch et al 2021 and available in the NCBI Taxonomy browser (https://www.ncbi.nlm.nih.gov/Taxonomy/Browser/wwwtax.cgi)
Starting with the high-level taxonomic units like Archaea, Bacteria, etc., all respective lower taxonomic units (i.e. kingdom, clade, phylum, family, etc.) were extracted by creating the appropriate taxon_id lists.
The following 10 top-level taxonomic units were used to retrieve the lower taxonomic units, in parentheses the names used on the left side of Figure 1 if different from the denomination of the respective tax_id identifier (Taxonomy ID):

- Archaea, Taxonomy ID: 2157
- Bacteria, Taxonomy ID: 2
- Viruses [not monophyletic] (Moreira et al. 2009), Taxonomy ID: 10239
- Other sequences (Others), Taxonomy ID: 28384
- Unclassified sequences (Unclassified), Taxonomy ID: 12908
- *Homo sapiens* (Human),Taxonomy ID: 9606
- Viridiplantae (Green plants),Taxonomy ID: 33090
- Fungi, Taxonomy ID: 4751
- Metazoa without *Homo sapiens (Non-human animals)*, Taxonomy ID: 33208 without 9606 (*Homo sapiens*)
- Other eukaryote [not monophyletic], Taxonomy ID: 2759 without 9606 (*Homo sapiens*), without 33090 (Viridiplantae), without 4751 (Fungi), and without 33208 (Metazoa)

The 10 tax_id lists were used to categorize each ENA accession to the appropriate taxonomic category.

- For 1.958.758 accessions (0.66% of the total), a taxonomic grouping could not be determined (e.g., because the tax_id field was empty, or the tax_id was obsolete) and thus these accessions have been included in the “Unclassified” group of Figure 1.

**Supplementary References**

Federhen, S. (2012). The NCBI Taxonomy database. *Nucleic Acids Research*, *40*(D1), D136–D143. https://doi.org/10.1093/nar/gkr1178

Moreira, D., & López-García, P. (2009). Ten reasons to exclude viruses from the tree of life. Nature reviews. Microbiology, 7(4), 306–311. https://doi.org/10.1038/nrmicro2108

Sakamoto, T., & Ortega, J. M. (2021). Taxallnomy: An extension of NCBI Taxonomy that produces a hierarchically complete taxonomic tree. *BMC Bioinformatics*, *22*(1), 388. https://doi.org/10.1186/s12859-021-04304-3

Schoch, C. L., Ciufo, S., Domrachev, M., Hotton, C. L., Kannan, S., Khovanskaya, R., Leipe, D., Mcveigh, R., O'Neill, K., Robbertse, B., Sharma, S., Soussov, V., Sullivan, J. P., Sun, L., Turner, S., & Karsch-Mizrachi, I. (2020). NCBI Taxonomy: A comprehensive update on curation, resources and tools. Database, 2020, baaa062. https://doi.org/10.1093/database/baaa062

Shen, W., & Ren, H. (2021). TaxonKit: A practical and efficient NCBI taxonomy toolkit. *Journal of genetics and genomics = Yi chuan xue bao*, *48*(9), 844–850. https://doi.org/10.1016/j.jgg.2021.03.006

Zaharia, M., Xin, R. S., Wendell, P., Das, T., Armbrust, M., Dave, A., Meng, X., Rosen, J., Venkataraman, S., Franklin, M. J., Ghodsi, A., Gonzalez, J., Shenker, S., & Stoica, I. (2016). Apache Spark: A unified engine for big data processing. *Communications of the ACM*, *59*(11), 56–65. https://doi.org/10.1145/293466
